# Fibroblast growth factor 10 alleviates particulate matter-induced lung injury by inhibiting the HMGB1-TLR4 pathway

**DOI:** 10.18632/aging.102676

**Published:** 2020-01-20

**Authors:** Lingjing Liu, Chenjian Song, Jingli Li, Qiang Wang, Mingyang Zhu, Yiran Hu, Junjie Chen, Chaolei Chen, Jin-San Zhang, Nian Dong, Chengshui Chen

**Affiliations:** 1 Department of Pulmonary Medicine, Wenzhou Medical University First Affiliated Hospital, Wenzhou 325006, China; 2 Department of Pulmonary Medicine, Yiwu Central Hospital, Yiwu 322000, China; 3 Department of Pharmacy, Wenzhou Medical University Pharmacy School, Wenzhou 325006, China

**Keywords:** particulate matter, FGF10, lung injury, inflammation, HMGB1, TLR4

## Abstract

Exposure to particulate matter (PM) is associated with increased incidence of respiratory diseases. The present study aimed to investigate the roles of fibroblast growth factor 10 (FGF10) in PM-induced lung injury. Mice were intratracheally instilled with FGF10 or phosphate-buffered saline at one hour before instillation of PM for two consecutive days. In addition, the anti-inflammatory impact of FGF10 *in vitro* and its effect on the high-mobility group box 1 (HMGB1)-toll-like receptor 4 (TLR4) pathway was investigated. It was found that PM exposure is associated with increased inflammatory cell infiltration into the lung and increased vascular protein leakage, while FGF10 pretreatment attenuated both of these effects. FGF10 also decreased the PM-induced expression of interleukin (IL)-6, IL-8, tumor necrosis factor-α and HMGB1 in murine bronchoalveolar lavage fluid and in the supernatants of human bronchial epithelial cells exposed to PM. FGF10 exerted anti-inflammatory and cytoprotective effects by inhibiting the HMGB1-TLR4 pathway. These results indicate that FGF10 may have therapeutic values for PM-induced lung injury.

## INTRODUCTION

Fine particulate matter (PM) is a prominent pollutant present in the air, and it represents a significant threat to public health, since a number of epidemiological studies have revealed that exposure to PM is associated with higher rates of asthma [1], chronic obstructive pulmonary disease [2], and lung cancer [3]. Perhaps most strikingly, short- or long-term exposure to fine particulate PM2.5 (<2.5 μm in diameter) is associated with increased morbidity and mortality [4–5]. Due to the small size, these PM2.5 particles can readily be inhaled and deposited throughout the airways. The deposition of these particles in the alveoli can allow these to readily access capillaries and enter circulation, thereby leading to injury to a wide number of different tissues and organs [6–7]. The inherently toxic nature of some of these absorbed compounds, which can include heavy metals, sulfates, endotoxins, or polycyclic aromatic hydrocarbons, can further exacerbate the associated toxicity [8]. According to the American Cancer Society, there is a 6% increase in cardiopulmonary mortality for each 10 μg/m^3^ increase in PM level [9]. Since it is difficult to control the environmental problems that drive the high PM level exposure, it is essential to identify alternative preventative or therapeutic approaches to protect the human respiratory system from PM-mediated respiratory injury.

Fibroblast growth factor 10 (FGF10) is a member of the paracrine FGF signaling molecules that signals through the high affinity fibroblast growth factor receptor 2 (FGFR2)-IIIb receptor and mediates a range of effects that are vital to the development of the lungs and other organs [10–12]. When *Fgf10* or *Fgfr2-IIIb* are fully knocked out in mice, this results in complete perinatal lethality and a complete lack of lung formation, while partial *Fgf10* deletion results in murine neonatal lethality attributable to lung hyperoxia exposure [13–15]. Recent studies have suggested that deficiencies in FGF10 can be a key driver for chronic lung diseases, such as chronic obstructive pulmonary disease [16–17], bronchopulmonary dysplasia [18], and idiopathic pulmonary fibrosis (IPF) [19]. At present, it remains uncertain whether the administration of exogenous FGF10 can reduce the impact of PM-induced injury on the respiratory system. Therefore, in the present study, a murine model of intratracheal PM instillation was used to explore the therapeutic efficacy of exogenous FGF10 administration.

Pattern recognition receptors (PRRs) have recently been identified as key mediators of airway cell inflammation in response to pollutant exposure [20]. There are many different types of PRRs that recognize unique types of ligands, which include toll-like receptors (TLRs), retinoic acid-inducible gene-I-like receptors, C-type lectin receptors, and nucleotide-binding oligomerization domain-like receptors [21–22]. These PRRs are capable of specifically recognizing certain conserved pathogen-associated molecular patterns, as well as endogenously derived injury-associated molecular patterns, released from cells under stress, and upon recognition of these signals, these PRRs activate the appropriate intracellular signaling to mediate cytokine production and alter gene expression [23]. TLR4 has been shown to be particularly relevant in the context of acute lung injury, mediating the acute lung injury that is associated with hemorrhagic shock or ventilator use. Similarly, TLRs have been found to be important for immunological responses to air pollution [24–25].

In addition to the potential pathogen-associated molecular patterns present in the context of air pollution, the release of injury-associated molecular patterns, which include the heat shock proteins and high-mobility group box 1 (HMGB1) from injured airway cells, can drive the PRR activation [26]. HMGB1 is a nuclear and cytoplasmic protein that is ubiquitously expressed in almost all cells, and is secreted into the extracellular environment in the context of inflammation. Recent studies have reported the association between FGF10 and HMGB1 in extrapulmonary tissues, and once present in circulation, HMGB1 can engage TLRs, including TLR4, and drive the expression of proinflammatory cytokines, including tumor necrosis factor (TNF)-α, interleukin (IL)-1β and IL-6 [27]. However, it remains unknown whether the association between FGF10 and HMGB1 also exists in the lungs.

In the present study, the present hypothesis was tested using a mouse model of PM-induced lung injury, and it was demonstrated that FGF10 could inhibit the pro-inflammatory response of lung tissues and human bronchial epithelial cells (HBECs) induced by PM, and improve the recovery of lung injury induced by PM by inhibiting the HMGB1-TLR4 pathway.

## RESULTS

### FGF10 reduced PM-induced lung injury

**Figure 1:**
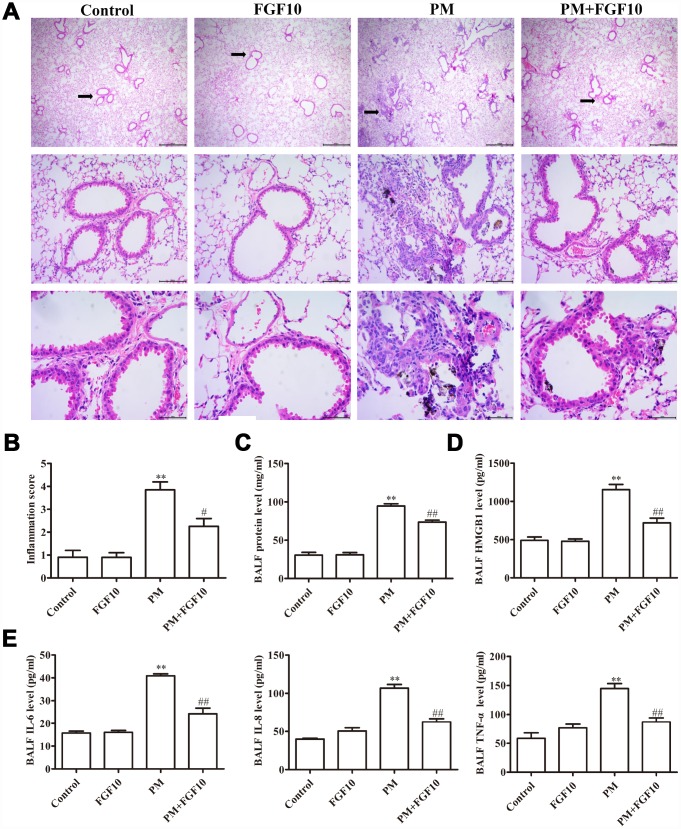
**Pretreatment with FGF10 reduced the inflammation in response to PM exposure *in vivo*.** Mice were intratracheally treated with 5 mg/kg of FGF10 solution at one hour in advance, after which these were intratracheally instilled with 100 μg of PM/day/mouse over two consecutive days. (**A**) The representative H&E stained lung sections. (**B**) The inflammatory scores for the H&E stained lung sections (*n*=3/group). (**C**) The total BALF protein as measured *via* the BCA assay. (**D** and **E**) The BALF HMGB1, IL-6, IL-8 and TNF-α protein levels as quantified by ELISA. Data were presented as mean ± standard error of the mean (SEM, *n*=3); *P<0.05, **P<0.01 *vs.* control. ^#^*P*<0.05, ^##^*P*<0.01 *vs.* PM.

When the structural injury was assessed in the lung samples obtained from different treatment groups (*n*=3 mice/group), it was found that PM exposure was associated with a marked increase in acute lung inflammation, when compared to the lungs of control mice. This inflammation was characterized by a significant increase in inflammatory cell accumulation in the airways and alveoli. In contrast, mice pretreated with FGF10 exhibited a significant reduction in inflammatory cell infiltration into the lungs (Figure 1A). Consistent with this, PM treatment was associated with a significant increase in inflammatory scores, when compared to control mice, while FGF10 pretreatment significantly reduced this PM-induced inflammatory score (Figure 1B). Furthermore, PM exposure was associated with a significant increase in bronchoalveolar lavage fluid (BALF) protein levels, when compared to control mice, while FGF10 pretreatment for one hour prior to PM exposure was associated to significantly reduced BALF protein levels in response to PM (Figure 1C). Furthermore, PM exposure was associated with a significant increase in BALF HMGB1 protein levels, while FGF10 pretreatment was sufficient to significantly reduce these levels upon PM stimulation (Figure 1D). Consistent with this, FGF10 pretreatment was associated with a significant reduction in the levels of IL-6, IL-8 and TNF-α in the BALF of PM-exposed mice (Figure 1E).

### PM increased airway epithelial p-FGFR2 and HMGB1 expression

**Figure 2:**
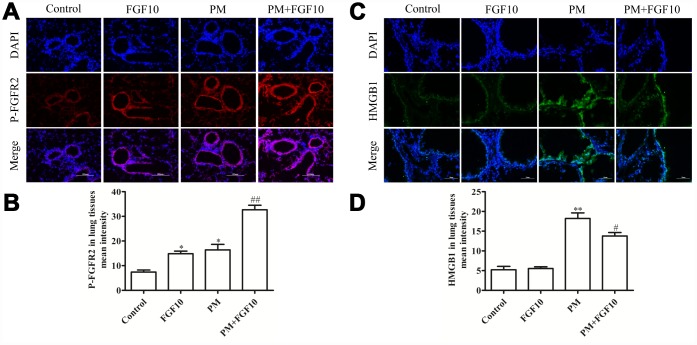
**The FGF10 pretreatment and PM treatment led to p-FGFR2 activation *in vivo*, while FGF10 reduced the HMGB1 expression**. Mice were intratracheally treated with 5 mg/kg of FGF10 solution at one hour in advance, after which these were intratracheally instilled with 100 μg of PM/day/mouse over two consecutive days. At two days post-PM exposure, the immunofluorescent detection of lung p-FGFR2 (**A** and **B**, scale bars = 100 μm) and HMGB1 (**C** and **D**) was conducted. DAPI (blue) was used for nuclear staining (scale bars = 50 μm).

The link between PM-induced lung injury and the FGF10/FGFR2 signaling pathway in mice was explored by assessing the FGFR2 receptor activation (p-FGFR2) *via* immunofluorescent staining. Compared to control mice, PM exposure was associated with a significant increase in p-FGFR2 expression in the airway epithelium, and this was further increased when mice were treated with FGF10 prior to PM exposure (Figure 2A and 2B). The HMGB1 levels in the lungs of these mice was assessed using the same experiment approach, revealing that FGF10 pretreatment is sufficient to attenuate a large part of the PM-induced increase in lung tissue HMGB1 expression (Figures 2C and 2D).

### FGF10 prevented PM-induced HBEC inflammation and cell death

**Figure 3:**
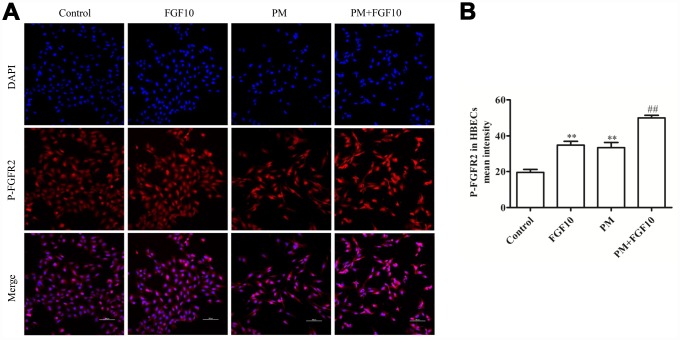
**The PM exposure and FGF10 pretreatment were associated with the p-FGFR2 activation in HBECs.** DAPI (blue) was used for nuclear staining. Scale bars=100 μm.

**Figure 4:**
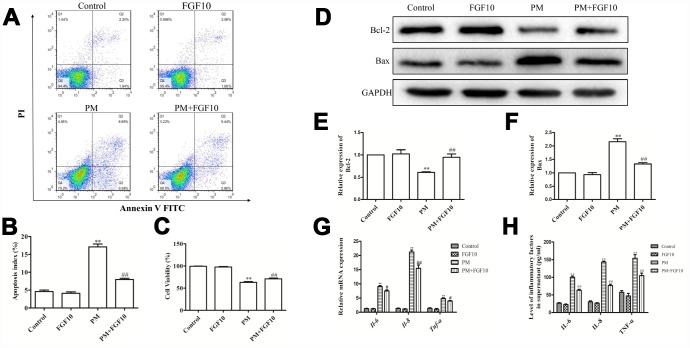
**FGF10 alleviated the cellular injury in response to PM.** (**A** and **B**) FGF10 (10 ng/ml) was used to pretreat HBECs for one hour, after which these were exposed for 24 hours to 200 μg/cm^3^ of PM. Apoptotic death was confirmed *via* flow cytometry. (**C**) The HBECs treated with FGF10 and/or PM were assessed for viability by CCK-8 assay. (**D**–**F**) The expression of Bcl-2 and Bax were detected by western blot, with GAPDH as the loading control. (**G**) The HBECs that were treated with FGF10 and/or PM were collected, and the IL-6, IL-8 and TNF-α mRNA levels were measured *via* RT-qPCR. (**H**) The supernatants obtained from HBECs that were treated with FGF10 and/or PM were collected, and the IL-6, IL-8 and TNF-α levels were quantified *via* ELISA. Data were presented as the mean ± standard error of the mean (SEM) of three independent experiments. *P<0.05, **P<0.01 *vs.* control. ^#^*P*<0.05, ^##^*P*<0.01 *vs.* PM.

In line with the present *in vivo* immunofluorescent staining results, the p-FGFR2 activation in HBECs upon PM exposure was observed (Figure 3A, 3B). When HBECs were treated with FGF10 prior to PM exposure, this significantly reduced the PM-induced apoptosis (Figure 4A, 4B) and significantly increased cell viability by 12.3%, when compared to that of the PM stimulation. Then, the expression of pro-apoptotic proteins involved in cell apoptosis, Bcl-2 and Bax were examined by western blot (Figures 4D–4F), and it was found that the expression of Bax significantly increased upon PM exposure in HBECs, while Bcl-2 expression decreased. It is significant that the FGF10 treatment inhibited the pro-apoptotic expression of Bax and Bcl-2. It was further confirmed that HBECs expressed high levels of IL6, IL8 and TNF-α at the mRNA level following PM treatment, while FGF10 pretreatment reversed this upregulation by 1.1-fold, 1.4-fold and 1.2-fold, respectively (*P*<0.05, Figure 4G). The same was true at the protein level, with FGF10 reducing the PM-induced increase in supernatant levels of IL-6, IL-8 and TNF-α (Figure 4H).

### FGF10 suppressed the HMGB1-TLR4 pathway

**Figure 5:**
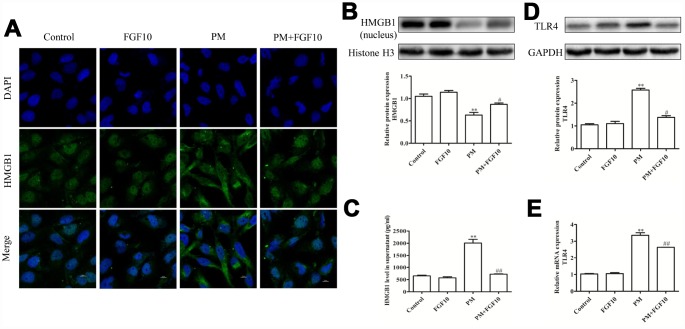
**FGF10 reduced the nuclear HMGB1 release and expression of TLR4.** (**A**) FGF10 (10 ng/ml) was used to pretreat HBECs for one hour, after which these were exposed for 24 hours in 200 μg/cm^3^ of PM or PBS. The HMGB1 expression was assessed *via* confocal microscopy. DAPI (blue) was used for the nuclear staining. Scale bars=10 μm. (**B**) The nuclear HMGB1 protein levels were assessed *via* western blot, and GAPDH was used as the loading control. (**C**) The supernatant HMGB1 levels were measured *via* ELISA. (**D** and **E**) The TLR4 expression in HBECs was assessed *via* western blotting and RT-qPCR. Data were presented as the mean ± standard error of the mean (SEM) of three independent experiments. *P<0.05, **P<0.01 *vs.* control. ^#^*P*<0.05, ^##^*P*<0.01 *vs.* PM.

In order to determine how FGF10 mediates the long-term protection of the lungs, confocal imaging of HMGB1 in cells was conducted following the *in vitro* PM-mediated injury. Briefly, the localization of DAPI and HMGB1 expression was compared, and it was revealed that PM exposure led to reduced nuclear HMGB1 levels and increased cytoplasmic and extracellular HMGB1 localization. However, the pretreatment with FGF10 reversed this effect and reduced levels of cytoplasmic and secreted HMGB1 (Figure 5A).

Next, nuclear proteins were isolated from differentially treated HBECs to measure the HMGB1 levels by western blot, while the supernatant levels of this protein were assessed by enzyme-linked immunosorbent assay (ELISA). These experiments confirmed that PM treatment is associated with the reduction in nuclear HMGB1 levels at 24 hours post-PM exposure, while the pretreatment with FGF10 reversed this effect, increasing the nuclear HMGB1 levels (Figure 5B). FGF10 also inhibited the PM-mediated HMGB1 release from these HBECs (Figure 5C). Given that HMGB1 is known to signal through TLR4 to drive inflammation, the TLR4 signaling in these cells were analyzed, and this revealed the increase in mRNA and protein levels of TLR4 following PM exposure, which was significantly attenuated by FGF10 (Figure 5D–5E). This suggests that FGF10 can inhibit the translocation of HMGB1 from the nucleus to the cytoplasm, thereby impeding any consequent inflammation induced through the HMGB1-TLR4 signaling axis.

### The overexpression of HMGB1 compromised the cytoprotective effect of FGF10

**Figure 6:**
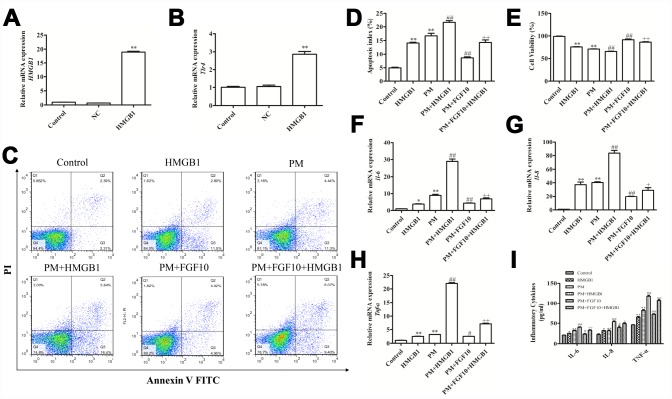
**The increase in HMGB1 levels compromised the cytoprotective effect of FGF10.** (**A** and **B**) The recombinant HMGB1 plasmid transfection upregulated the HMGB1 expression and TLR4 activation. (**C**–**E**) HMGB1-overexpressing cells were treated with FGF10, after which apoptotic death and viability were measured following PM exposure. (**F**–**H**) The IL-6, IL-8 and TNF-α levels were measured *via* RT-qPCR. (**I**) The supernatant levels of IL-6, IL-8 and TNF-α obtained from the treated cells were quantified *via* ELISA. Data were presented as the mean ± standard error of the mean (SEM) of three independent experiments. *P<0.05, **P<0.01 *vs.* control. ^#^*P*<0.05, ^##^*P*<0.01 *vs.* PM. ^+^*P*<0.05, ^++^*P*<0.01 *vs.* PM+FGF10.

In order to determine how FGF10 regulates HMGB1 and protects from PM-induced lung injury, HBECs cells were transfected with the recombinant *pcDNA-HMGB1* plasmid to overexpress HMGB1 (Figure 6A). Consistent with the increase in *HMGB1* expression in these cells, there was an increase in TLR4 expression upon HMGB1 overexpression, thereby confirming the functional relevance of this phenotype (Figure 6B). Importantly, it was found that the ability of FGF10 to protect cells against PM-induced cytotoxicity was significantly reduced upon HMGB1 overexpression, which also promoted apoptosis (Figure 6C, 6D) and inhibited cell proliferation (Figure 6E). Similarly, it was found that the HMGB1 overexpression reduced the ability of FGF10 to decrease the PM-induced apoptosis and mRNA and protein levels of IL-6, IL-8 and TNF-α (Figures 6F–6I).

### HMGB1 knockdown attenuated the PM-induced cellular inflammation

**Figure 7:**
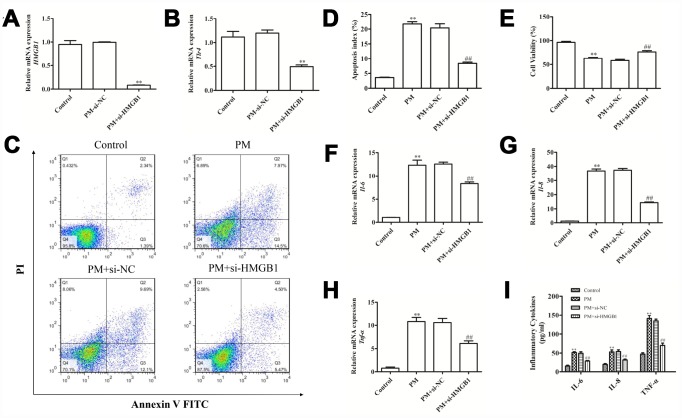
**Disrupting the HMGB1-mediated signaling reduced the apoptotic death and inflammation induced by PM.** (**A** and **B**) HBECs were exposed to PM after si-HMGB1 transfection, and RT-qPCR was used to assess the HMGB1 and TLR4 expression. (**C**–**E**) The PM-induced apoptosis and viability effect were analyzed following HMGB1 knockdown in cells. (**F**–**H**) HBECs were exposed to PM after si-HMGB1 transfection, and RT-qPCR was used to assess the IL-6, IL-8 and TNF-α expression. (**I**) HBECs were exposed to PM after si-HMGB1 transfection, and the IL-6, IL-8 and TNF-α levels in the supernatant were measured *via* ELISA. Data were presented as the mean ± standard error of the mean (SEM) of three independent experiments. *P<0.05, **P<0.01 *vs.* control. ^#^*P*<0.05, ^##^*P*<0.01 *vs.* PM.

An siRNA was used to knockdown *HMGB1* in HBECs. This knockdown significantly reduced the *HMGB1* mRNA level at 24 hours post-PM exposure (Figure 7A), with a simultaneously decreased TLR4 expression in these *HMGB1-*knockdown cells (Figure 7B). This suggests that HMGB1 mediated the upregulation of TLR4 in HBECs following PM exposure. Furthermore, the HMGB1 knockdown improved the HBEC apoptosis (Figure 6C, 6D), promoted the cell proliferation (Figure 6E) in response to PM exposure, and attenuated the PM-induced upregulation of mRNA and protein levels of IL-6, IL-8 and TNF-α (Figure 7F–7I).

## DISCUSSION

The present study demonstrated that PM could induce the expression of molecules associated with inflammation and the destruction of lung tissue integrity, including IL-6, IL-8 and TNF-α. Furthermore, PM exposure lead to the nucleocytoplasmic translocation of HMGB1 and the activation of the HMGB1-TLR4 pathway, which is attenuated by FGF10. Using anti-HMGB1 siRNA, it was demonstrated that PM-induced airway epithelial cell injury and inflammation was alleviated through HMGB1-TLR4 signal transduction. Using a recombinant pcDNA-HMGB1 plasmid to increase the protein expression of HMGB1, it was found that HMGB1 overexpression compromised the protective effect of FGF10 against PM-induced cell inflammation.

There is close relationship between exposure to PM *via* air pollution and the increase in respiratory and cardiovascular morbidity and mortality [28–29]. Inflammation has been considered to be the primary mediator of PM-induced respiratory injury [30]. PM-associated inflammation is responsible for the increased production of IL-1β, IL-6, IL-8, IL-10 and TNF-α by airway epithelial cells [31–33], suggesting that the monitoring of these cytokines may be an effective means of gauging the severity of PM-induced inflammation. In the present study, it was found that PM exposure led to airway inflammatory cell infiltration, changes in lung morphology, and increased levels of IL-6, IL-8, TNF-α and HMGB1 in the BALF, suggesting the successful modeling of PM-induced lung injury.

Studies on FGF10 in lung cancer have reveal that unidirectional FGF signaling safeguards normal physiological conditions against morbid autocrine stimulation, and the importance of the FGF signaling axis has been highlighted by the ligand-promiscuous stimulation and ectopic expression of FGFRs in pathological settings, including cancer stem cells [34]. A recent study on small cell lung cancer patients identified FGF10 amplification in 37.5% of patients and FGFR1 amplification in 25% of patients [35]. FGF10 plays an important role in both tissue repair and in controlling the mesenchymal to epithelial transition during organ development [36]. The partial or complete knockout of FGF10 in mice can lead to serious developmental problems of the lung and other organs [37–38]. These lung-specific functions of FGF10 may provide further insight into the molecular mechanisms by which FGF10, but not other FGFs with binding affinity for FGFR1b, affects cancer stem cells derived only from the lung. Another study revealed that FGF10 from mesenchyme-derived mesenchymal stromal cells preferentially activates the epithelial-type FGFR1b of lung cancer cells, resulting in the reduction of cancer stem cells [39]. Previous studies have also shown that a recombinant form of FGF10 has no effect on the growth of a certain lung cancer cell line, and that the forced overexpression of FGF10, rather than a disrupted lung morphogenesis, causes pulmonary adenomas [40–41]. In addition, it has been shown that FGF10 prevents lung injury and promotes lung epithelial regeneration after various stresses, including influenza-induced acute respiratory distress syndrome [42], lipopolysaccharide-induced lung injury [43], and mechanical ventilation-induced lung injury [44]. The overexpression of a dominant-negative Fgfr2 receptor, specifically in the lung epithelium, inhibits the retinoic acid-induced alveolar regeneration in association with the increase in PDGFRαpos and reduced expression of SMA in interstitial myofibroblasts [45]. Similarly, FGF10 attenuates water-induced alveolar epithelial DNA damage *via* the Grb2-SOS/Ras/Raf-1/MAPK pathway [46]. Lung resident mesenchymal stromal cells isolated from FGF10-pretreated rats are protected against lipopolysaccharide-induced acute lung injury [47]. It has also been suggested that FGF10 might have a potential therapeutic effect for lung edema due to its upregulating Na^+^/K^+^-ATPase activity in alveolar epithelial cells *via* the ERK1/2 pathway [48]. However, the mechanisms underlying these protective effects of FGF10 signaling during injury and the regeneration in adult lungs have not been fully elucidated. A previous study conducted by the investigators revealed that PM exposure increased the level of endogenous FGF10, suggesting the role of the endogenous FGF10/FGFR2 signaling pathway in the repairment of lung injury. In order to investigate the activation of FGFR by FGF10 in this model, the expression of activated FGFR was analyzed using the p-FGFR antibody. Consistent with the previous findings of the investigators, it was found that FGFR2 was mildly and locally activated in the airway epithelium after PM exposure. Compared to the PM group, the FGF10 treatment significantly increased the level of p-FGFR2. These present findings strongly suggest that the protective effect of FGF10 against lung injury is mediated by FGFR activation. Furthermore, the treatment with FGF10 before intratracheal PM administration in mice reduced the PM-induced lung injury and proinflammatory cytokine production. Furthermore, FGF10 further attenuated the PM-induced increase in BALF protein levels, suggesting that this growth factor might reduce capillary permeability and associated lung tissue edema *in vivo*. Consistently, these present *in vitro* findings revealed that the PM treatment increased the inflammatory cytokine production in these cells, which was attenuated by FGF10.

HMGB1 is one of the most well-understood injury-associated molecular patterns, serving as a proinflammatory molecule that can drive local inflammation when extracellularly exposed in response to stress. This extracellular release of HMGB1 can occur both passively *via* protein release from the injured cells and actively *via* direct HMGB1 secretion from live cells [49–51]. Then, the free HMBG1 binds to TLR2, TLR4 and the receptor for advanced glycation end products, thereby driving the upregulation of multiple proinflammatory factors, including TNF-α and IL-6 [52]. TLR4 is well-known as a PRR involved in acute lung injury of respiratory infection [53–54]. More recent studies have suggested that TLR4 is also an important mediator of noninfectious lung injury [55–56]. It was found that the HMGB1-TLR4 signaling pathway controls the inflammatory cytokine production and apoptotic death of cardiomyocytes in the context of myocardial ischemia/reperfusion injury [57]. HMGB1 has also been shown to drive the alveolar macrophage production of inflammatory factors, including IL-1β and TNF-α, thereby inducing acute lung injury [58]. Significantly elevated levels of HMGB1 were observed in the BALF of mice following PM exposure. In addition, HMGB1 was primarily in the nuclei in HBECs at baseline, while upon PM exposure, this was present at lower levels in the nuclei, and at higher levels in the cytoplasm and extracellular environments. These findings, together with the increase in TLR4 expression in response to PM, suggests that PM induced the HMGB1 translocation from the nucleus to the cytoplasm, resulting in its secretion and subsequent activation of TLR4. The HMGB1-TLR4 signaling was associated with inflammatory cell injury. In the present study, the activation of the HMGB1-TLR4 pathway promoted the production of IL-6, IL-8 and TNF-α, and caused cell damage. The pretreatment with FGF10 attenuated the HMGB1 upregulation in the BALF and lung tissues by suppressing its cytoplasmic translocation and secretion in response to PM. It also reduced the associated increase in TLR4 activation and the expression of IL-6, IL-8 and TNF-α. All these results revealed the potent protective effect of FGF10 against PM-induced lung injury.

The airway epithelium has been shown to be a primary source of abnormal HMGB1 expression in chronic airway disease, driving progressive inflammation in asthma and chronic obstructive pulmonary disease [59]. It has been found that HMGB1 is associated with increased airway smooth muscle contraction and TLR4 activation in asthma [60]. Neutralizing HMGB1 can attenuate asthma-associated airway remodeling and inflammation [61]. HMGB1 downregulation has been shown to protect against cell injury in multiple models of lung injury [62]. In the present study, the knockdown of *HMGB1* attenuated the PM-induced upregulation of inflammatory cytokines and TLR4, suggesting that HMGB1 is a key mediator of PM-induced inflammation. It was also found that HMGB1 overexpression compromised the protective effect of FGF10 against PM-induced cell injury.

The present study has limitations. First, the PM suspension, rather than the nebulized PM, was intratracheally administered to establish the mouse model, which may lack similarity to the natural condition of human diseases caused by air pollution. Second, the hemodynamic effect of PM exposure on respiratory injury was not considered in the present study, which may require an extended observation time.

## CONCLUSION

Exogenous FGF10 can protect against PM-mediated lung injury in mice by reducing the activation of the HMGB1-TLR4 signaling. These present findings suggest the therapeutic potential of FGF10 in preventing or treating PM-mediated lung injury.

## MATERIALS AND METHODS

### Reagents

The PM (1649b) was obtained from the Standard Reference Material Program, and this material is certified by the National Institute of Standards and Technology (MD, USA). The PM used in the present study primarily comprised of common components of urban PM, including pesticides, dioxins, polycyclic aromatic hydrocarbons and polychlorinated biphenyl congeners. The PM was used for murine intratracheal instillation [63]. The recombinant human FGF10 was obtained from PeproTech (Shanghai, China). The anti-HMGB1, anti-TLR4 and anti-GAPDH were obtained from Cell Signaling Technology (MA, USA). The anti-pFGFR2 was obtained from Affinity Biosciences. Beyotime (Shanghai, China) was the source of all other western blot reagents. The ELISA kits for defined antigens (IL-6, IL-8, TNF-α and HMGB1) were obtained from Boyun Biotechnology (Shanghai, China). The real-time polymerase quantitative chain reaction (RT-qPCR) primers were obtained from Sangon Biotech (Shanghai, China). All other RT-qPCR reagents were obtained from Takara Bio (Shiga, Japan).

### Animal model of PM-induced lung injury

All animal studies were conducted under the oversight of the Institutional Animal Care and Use Committee of Wenzhou Medical University (Wenzhou, China). In the present study, male C57BL/6 mice (*n*=40, 20–25 g) were utilized and obtained from the Experimental Animal Center of Wenzhou Medical University. These animals were housed in a standard 22–24°C specific pathogen-free environment, with free food and water access, and 2–3 mice per cage. Then, these animals were randomized into four treatment groups: (1) control group, treated with phosphate-buffered saline (PBS), (2) FGF10 group, (3) PM group, and (4) PM+FGF10 group. A 5-mg/kg FGF10 solution was intratracheally administered [64–65], while the control mice received saline solution. At one hour after these administrations, the PM (4 mg/kg) was intratracheally instilled to the model lung injury, while the control mice were instilled with PBS. After two consecutive days of PM instillation, these mice were sacrificed, and the lung tissue and BALF were collected.

### Cell culture

The HBECs were obtained from the Chinese Academy of Sciences (Shanghai, China), and grown in RPMI-1640 medium (Hyclone, UT, USA) containing 10% fetal bovine serum (Gibco, MA, USA) and penicillin/streptomycin (Gibco) at 37°C with 5% CO_2_. FGF10 (10 ng/ml) was added to appropriate cells for one hour, after which PM (200 μg/mL) or PBS was added for 24 hours. Then, the RNA and protein samples were collected for RT-qPCR and western blot, respectively.

### BALF collection

Mice were euthanized *via* 4% chloral hydrate, and eyeball extraction was conducted to mediate exsanguination. Then, the BALF was collected with a tracheal cannula, with 1 mL of PBS being gradually instilled into and withdrawn from the lung for three times. After collection, the BALF samples were centrifuged at 12,000 rpm for 15 minutes at 4°C.

### BALF protein measurement

A bicinchoninic acid (BCA) kit (Bio-Rad Laboratories) was used to quantify the amount of protein in the BALF samples, and a microplate reader (Molecular Devices, USA) was used to read the absorbance values at 570 nm.

### Hematoxylin and eosin staining (H&E)

After collection, 4% paraformaldehyde was used to fix the lung tissues. Then, these were paraffin-embedded and prepared into 5-μm slices. Afterwards, these sections were de-paraffinized, rehydrated, H&E stained, and analyzed using a light microscope. Three blinded investigators independently scored the degree of peribronchial and perivascular inflammation: 1, no inflammation was observed; 2, occasional cuffing with inflammatory cells; 3, most bronchi or vessels were surrounded by a thin layer (1 to 5) of inflammatory cells; 4, most bronchi or vessels were surrounded by a thick layer (>5) of inflammatory cells.

### Lung immunofluorescent staining

The lung sections were prepared as above. Then, the rehydrated tissue sections were probed for one hour with anti-HMGB1 and anti-phospho-FGFR2. Afterwards, the samples were washed in PBS and stained with Alexa Fluor 488-conjugated secondary antibodies for one hour. Then, DAPI was used for nuclear staining for 10 minutes, followed by fluorescent microscopic imaging.

### Cell viability measurement

Next, 1.5×10^4^ cells/well were plated in 100 μl of 96-well plates, and CCK-8 assay was performed to assess the cell viability. Briefly, cells that were treated with the appropriate siRNA or plasmid constructs and exposed to PM were used for this assay, with 10 μl of CCK-8 developing solution was added per well at 37°C for two hours. Then, the absorbance at 450 nm was determined using a spectrophotometer (Tecan, Männedorf, Switzerland).

### Cell apoptosis measurement

The apoptosis was quantified using the dual Annexin V/Propidium iodide (PI) staining. Briefly, the treated cells were resuspended in a 200-μl binding buffer volume, and 5 μl of Annexin V-FITC and 5 μl of PI (Beyotime) were added. Then, these cells were stained with this solution for 15 minutes protected from light. Afterwards, a fluorescent activated cell sorting flow cytometer (BD Biosciences, CA, USA) was used to quantify the apoptotic cells.

### RT-qPCR

TRIzol (Invitrogen, CA, USA) was used to isolate the cellular RNA, and the cDNA was prepared based on the provided protocols. The relative expression of murine IL-6, IL-8, TNF-α and TLR4 was quantified *via* the ΔCt approach, and GAPDH was used for normalization. These samples were analyzed in triplicate, with the primers used, as follows: IL-6 (sense, 5′-TTCGGTCCAGTTGCCTTCT-3′; *anti-sense*, 5′-GGTGAGTGGCTGTCTGTGTG-3′), IL-8 (sense, 5′-TTGCCAAGGAGTGCTAAAGAA-3′; *anti-sense*, 5′-TTGCCAAGGAGTGCTAAAGAA-3′), *TNF-α* (5′-AGCTGGTGGTGCCATCAGAGG-3′; *anti-sense*, 5′-TGGTAGGAGACGGCGATGCG-3′), TLR4 (sense, 5′-GACTGGGTAAGGAATGAGCTAG-3′; *anti-sense*, 5′-ACCTTTCGGCTTTTATGGAAAC-3′), and GAPDH (sense, 5′-AGGTCGGTGTGAACGGATTTG-3′; *anti-sense*, 5′-TGTAGACCATGTAGTTGAGGTCA-3′).

### Western blot

RIPA supplemented with phenylmethanesulfonyl fluoride (Beyotime) and phosphatase inhibitors (Biotool, TX, USA) was used to extract the cellular proteins, after which a BCA kit was used for the quantification of protein content, as above. Then, identical protein amounts (30 μg) were separated *via* SDS-PAGE and transferred onto polyvinylidene fluoride (PVDF) membranes. Afterwards, these membranes were blocked for one hour before overnight incubation with the appropriate primary antibodies (1:1,000) at 4°C. Then, these samples were washed for three times with TBST and probed with the appropriate HRP-linked secondary antibodies (1:5,000) for one hour at room temperature. Afterwards, these blots were washed again, and the protein was visualized *via* electrochemiluminescence, in which an X-ray film was used for exposure. The densitometry analyses of the resultant protein bands were conducted using the Molecular Analyst software (Biorad Laboratories, CA, USA). GAPDH served as a loading control.

### ELISA

The levels of IL-6, IL-8, TNF-α and HMGB1 in the BALF and the supernatant samples were quantified using the indicated ELISA kits, following the provided protocols for all analyses.

### HMGB1 knockdown

In order to knockdown the HMGB1 expression, siRNA constructs obtained from Hanbio Biotechnology Co., Ltd. (Shanghai, China) were used. The negative control (NC) siRNA: sense strand: 5′-UUCUCCGAACGUGUCACGUdTd-3′ and antisense strand: 5′-ACG UGACACGUUCGGAGAAdTd-3′; *HMGB1* siRNA: sense strand: 5’-GGAGAGAUGUGGAAUAACAdTd-3′ and antisense strand: 5′-UGUUAUUCCACAUCUCUCCdTd-3′. A total of 50 nM of these constructs were transfected into appropriate cells with Lipofectamine 2000 (Invitrogen). Briefly, the constructs were prepared for 20 minutes in Lipofectamine, after which these were added to cells for 48 hours at 37°C, and RT-qPCR was used to confirm the successful knockdown.

### HMGB1 overexpression

In order to achieve the *HMGB1* overexpression, the cDNA encoding the entirety of the *HMGB1* was cloned into the pcDNA 3.1 vector (Invitrogen). Then, HBECs were grown until 70-80% confluent, after which Lipofectamine 2000 (Invitrogen) was used to transfect these cells with 0.8 μg of recombinant or empty control vector.

### Confocal microscopy

Next, 1×10^5^ cells/well were plated onto 18-mm glass coverslips for 24 hour, after which cells were treated with FGF10 and PM, as above. Then, the media was removed, and the cells were washed and treated for 15 minutes with 0.1% Triton X-100 in PBS at room temperature. Afterwards, 10% goat serum was used to block the cells for one hour, followed by incubation with the primary anti-HMGB1 primary or anti-P-FGFR2. Then, Alexa 488-546 conjugated secondary antibodies were used for antigen detection. Subsequently, DAPI was used for nuclear staining, after which a confocal microscope (Leica Microsystems, Wetzlar, Germany) was used to image the cells.

### Statistical analysis

All data were presented as the mean ± standard deviation of three independent experiments. SPSS 19.0 (SPSS Inc., IL, USA) was used for all statistical tests. One-way ANOVA was used to compare the differences between groups, with *P*<0.05 as the significance threshold.
